# A novel zinc(II) complex with the ligand 2,2′,2′′-(1,4,7-triazanonane-1,4,7-triyl)triacetate (NOTA)

**DOI:** 10.1107/S1600536808041895

**Published:** 2008-12-17

**Authors:** Iria Pereira-García, Alejandro Macías, Rufina Bastida, Laura Valencia

**Affiliations:** a Departamento de Química Inorgánica, Facultade de Química, Avd. das Ciencias s/n, Universidade de Santiago de Compostela, 15706-Santiago de Compostela, A Coruña, Galicia, Spain

## Abstract

The zinc(II) complex with NOTA [2,2′,2′′-(1,4,7-triazanonane-1,4,7-triyl)triacetate] has previously been synthesized and studied in solution, but was not isolated. The corresponding title Zn^II^ complex penta­sodium(I) bis­{[2,2′,2′′-(1,4,7-triazanonane-1,4,7-triyl)triacetato]zinc(II)} tris­(perchlorate) methanol solvate, Na_5_[Zn(C_12_H_18_N_3_O_6_)]_2_(ClO_4_)_3_·CH_3_OH, was crystallized as a sodium perchlorate double salt in methano­l solution. The asymmetric unit contains two independent [Zn(NOTA)]^−^ complex anion entities, five sodium cations, three perchlorate anions and a methanol solvent mol­ecule. The two Zn^II^ cations exhibit a distorted trigonal-prismatic N_3_O_3_ coordination with a bifacial arrangement of the donor atoms. Neither the methanol solvent mol­ecule nor the perchlorate anions appear to be coordinated to the Zn centres. The crystal structure shows a layer arrangement parallel to (001) generated by inter­actions between the [Zn(NOTA)]^−^ units, the Na^+^ cations, two ClO_4_^−^ units and the methanole mol­ecule, leading to an overall layer composition of [Na_5_[Zn(C_12_H_18_N_3_O_6_)]_2_(ClO_4_)_2_^.^CH_3_OH]^+^. The third ClO_4_ anion is isolated and situated between the layers without any significant inter­actions.

## Related literature

Details on the synthesis of NOTA are given by Desreux (1980 ▶). For NOTA complexes of Al, Cr, Fe, Co, Ni, Cu, Ga and In characterized by X-ray diffraction studies, see: Boeyens & Van der Merwe (1997 ▶); Bossek *et al.* (1995 ▶); Clarke & Martell (1991 ▶); Craig *et al.* (1989 ▶); Jyo *et al.* (1990 ▶); Van der Merwe *et al.* (1983 ▶, 1985 ▶); Moore *et al.* (1990 ▶); Wieghardt *et al.* (1982 ▶). For general background, see: Geraldes *et al.* (1985 ▶).
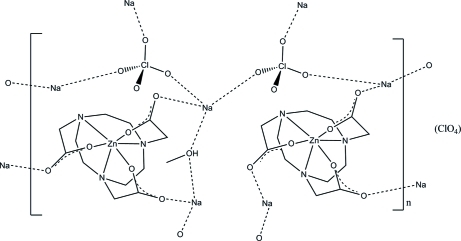


## Experimental

### 

#### Crystal data

Na_5_[Zn(C_12_H_18_N_3_O_6_)]_2_(ClO_4_)_3_·CH_4_O
*M* *_r_* = 1176.67
Orthorhombic, 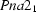

*a* = 16.8879 (5) Å
*b* = 9.4723 (3) Å
*c* = 26.4552 (9) Å
*V* = 4232.0 (2) Å^3^
*Z* = 4
Mo *K*α radiation
μ = 1.47 mm^−1^
*T* = 100 (2) K
0.22 × 0.10 × 0.10 mm

#### Data collection

Bruker APEXII CCD diffractometer
Absorption correction: multi-scan (*SADABS*; Sheldrick, 1996 ▶) *T*_min_ = 0.738, *T*_max_ = 0.867
31309 measured reflections
9954 independent reflections
8605 reflections with *I* > 2σ(*I*)
*R*_int_ = 0.036

#### Refinement

*R*[*F* ^2^ > 2σ(*F* ^2^)] = 0.039
*wR*(*F* ^2^) = 0.084
*S* = 1.07
9954 reflections
601 parameters
1 restraint
H atoms treated by a mixture of independent and constrained refinement
Δρ_max_ = 0.56 e Å^−3^
Δρ_min_ = −0.58 e Å^−3^
Absolute structure: Flack (1983 ▶), 4585 Friedel pairs
Flack parameter: 0.383 (7)

### 

Data collection: *APEX2* (Bruker, 2005 ▶); cell refinement: *APEX2*; data reduction: *SHELXTL* (Sheldrick, 2008 ▶); program(s) used to solve structure: *SIR92* (Altomare *et al.*, 1993 ▶); program(s) used to refine structure: *SHELXL97* (Sheldrick, 2008 ▶); molecular graphics: *ORTEP-3 for Windows* (Farrugia, 1997 ▶); software used to prepare material for publication: *WinGX* (Farrugia, 1999 ▶).

## Supplementary Material

Crystal structure: contains datablocks I, global. DOI: 10.1107/S1600536808041895/wm2202sup1.cif

Structure factors: contains datablocks I. DOI: 10.1107/S1600536808041895/wm2202Isup2.hkl

Additional supplementary materials:  crystallographic information; 3D view; checkCIF report

## Figures and Tables

**Table 1:** Selected bond lengths (Å)

Zn1—O5	2.027 (3)
Zn1—O1	2.062 (2)
Zn1—O3	2.066 (2)
Zn1—N2	2.160 (3)
Zn1—N3	2.172 (3)
Zn1—N1	2.189 (3)
Zn2—O23	2.047 (3)
Zn2—O21	2.057 (3)
Zn2—O25	2.072 (3)
Zn2—N22	2.198 (3)
Zn2—N21	2.201 (3)
Zn2—N23	2.201 (3)

**Fig. 1.**
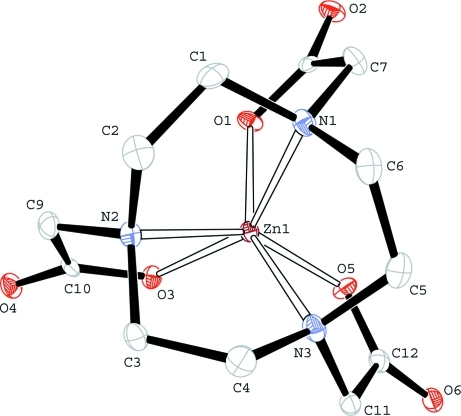
The molecular structure of one [Zn(NOTA)]- complex with atom labelling and displacement ellipsoids drawn at the 50% probability level. The structure of the second [Zn(NOTA)]- entity is very similar.

**Fig. 2.**
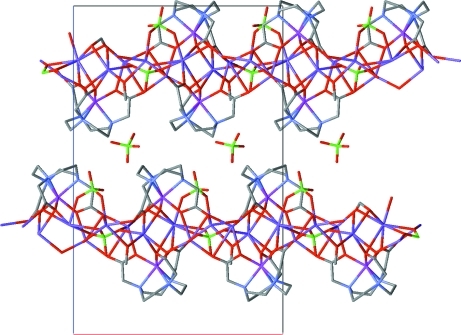
The crystal structure of the title compound in a projection along [010], emphasizing the layer arrangement parallel (001) as generated by interactions between the [Zn(NOTA)]- units, the Na+ cations, two ClO4- units and the methanol molecule. The third ClO4 anion is isolated and situated between the layers without any significant interactions.

## supplementary crystallographic information

### Comment

NOTA (1,4,7-triazacyclononane-N, N', N''-triacetate) has a well-known preference for small metal ions, and many metal NOTA complexes (*M* = Al, Cr, Fe, Co, Ni, Cu, Ga and In) have been structurally characterized: (Boeyens & Van der Merwe, 1997; Bossek *et al.*, 1995; Clarke & Martell, 1991; Craig *et al.*, 1989; Jyo *et al.*, 1990; Van der Merwe *et al.*, 1983, 1985; Moore *et al.*, 1990; Wieghardt *et al.*, 1982).

Our starting objective was the synthesis of NOTA (1,4,7-triazacyclononane-*N*,*N*',*N*''-triacetate) complexes in aqueous solutions and its isolation as salts of the type [*X*][*M*(NOTA)], where *X*^+^ is a monovalent cation and *M* is Zn^II^ or Cd(II). We also developed a species distribution diagram for NOTA complexes in aqueous solution based on the experimental data obtained by Geraldes *et al.* (1985). From these experiments we concluded that the anionic salts [Zn(NOTA)]^-^ and [Cd(NOTA)]^-^, due to their extreme solubility in aqueous solution, do not precipitate as neither sodium nor the alkylammonium salts. These difficulties for isolating the complexes led us to synthesize the Zn^II^ and Cd(II) NOTA complexes in methanol, a solvent in which the sodium salts are less soluble than in aqueous solution. A Zn^II^ complex with a 1:1 composition was prepared by reaction of the NOTA ligand *L* with hydrous Zn^II^ perchlorate in a 1:1 molar ratio of metal:ligand. This complex was synthesized by a single-step procedure as described and the reaction revealed a pure product that was also characterized by ESI-MS and ^1^H-NMR spectroscopy.

The molecular structure of the complex entity [Zn(NOTA)]^-^ and selected bond lengths (Å) and angles (°) of the coordination environment of Zn^II^ are given in Fig. 1 and Table 1, respectively. The asymmetric unit contains two independent mononuclear complex [Zn(NOTA)]^-^ entities, five sodium cations, three perchlorate anions and a methanol solvent molecule. The coordination environment, distances and angles of both independent [Zn(NOTA)]^-^ molecules are similar. When the metal centre coordination requirements do not favour an octahedral environment, the metal core geometry in NOTA complexes is trigonal-prismatic (Wieghardt *et al.*, 1982). Thus, the Zn^II^ centres present a six-coordinated N_3_O_3_ core in a distorted trigonal-prismatic arrangement. Each Zn atom is bound to three N atoms from the macrocyclic backbone and three O atoms from the pendant-arms. Like in all the other known structures of NOTA complexes, in the [Zn(NOTA)]^-^ entities the donor atoms are disposed in a bifacial arrangement. Three N atoms occupy one facial plane of the prism, and three O atoms belong to the other plane. The average Zn—N and Zn—O bond lengths are 2.187 Å and 2.055 Å, respectively. These bond lengths are in the range found for *M*–N and *M*–O bonds in other NOTA complexes with divalent transition metals.

The crystal structure shows a layer arrangement parallel (001) generated by interactions between the [Zn(NOTA)]^-^ units, the Na^+^ cations, two ClO_4_^-^ units and the methanol molecule, leading to an overall layer composition of [Na_5_[Zn(C_12_H_18_N_3_O_6_)]_2_(ClO_4_)_2_^.^CH_3_OH]^+^. The third ClO_4_ anion is isolated and situated between the layers without any significant interactions (Fig. 2).

### Experimental

Synthesis of the macrocycle NOTAH_3_: The ligand NOTA was prepared from its triazamacrocycle precursor TACN by alkylation with bromoacetic acid using a modification of a previously reported method (Desreux, 1980). TACN and NaOH were dissolved in water, and to the solution was added a bromoacetic acid/ NaOH aqueous solution at 273 K. The reaction mixture temperature was raised to 323 K, and a NaOH aqueous solution was added. The mixture was maintained at 323 K under stirring for 5 d. Then, concentrated hydrobromic acid was added until a pH of ~ 7 was reached. NOTAH_3_ does not precipitate from aqueous solutions in a well-defined state, thus a purification stage was needed. After a liquid-liquid extraction with n-butanol, a white powder characterized as a salt of the expected ligand was finally obtained. C_12_H_21_N_3_O_6_.(CH_2_BrCOOH)_3_:MS (ESI, *m/z*) 304 [H(LH_3_)]^+^, 326 {Na(LH_3_)]}^+^, 348 [Na_2_(LH_2_)]^+^; ^1^H NMR data: (250 MHz, D_2_O, SiMe_4_): d 3.5 (s, 12 H from –CH_2_– in the ring), d 3.9 (s, 6 H from CH_2_BrCOOH), d 4.3 (s, 6 H from –CH_2_– in pendants).

Synthesis of the metal complexes: Hydrated zinc perchlorate was added to a solution of the purified ligand NOTAH_3_ in methanol. The reaction mixture was heated and then cooled. A concentrated NaOH methanolic solution was added until a pH of 7 was reached. Single crystals were obtained by the diffusion vapour-phase crystallization method in a MeOH/Et_2_O solvent system. The Zn^II^ complex was characterized by ESI-MS, ^1^HNMR, COSY NMR and X-ray diffraction. Na[ZnL]1.5(NaClO_4_).0.5MeOH: MS(ESI, *m/z*) 366 [Zn(LH_2_)]^+^, 388 {Na[Zn(LH)]}^+^. Colour: colourless.

The correspondent Cd(II) complex was also obtained in methanolic solution, but it has not been isolated in crystalline form.

### Refinement

The absolute structure parameter was refined (Flack, 1983) and points to racemic twinning, with a ratio of the twin fractions of approximately 3:2. The hydrogen atoms attached to the carbon atoms were located in their calculated positions and refined using a riding model with U(H) equal to 1.2× U_eq_ (1.5 for methyl groups) of the parent atom and C—H= 0.97 Å. The hydrogen atom attached to the oxygen atom in the methanol molecule was localized in a Fourier map and refined with U_iso_ constrained to be 1.5 × U_eq_ of the O atom.

### Crystal data

**Table:**

Na_5_[Zn(C_12_H_18_N_3_O_6_)]_2_(ClO_4_)_3_·CH_4_O	*F*(000) = 2392
*M**_r_* = 1176.67	*D*_x_ = 1.847 Mg m^−^^3^
Orthorhombic, *P**n**a*2_1_	Mo *K*α radiation, λ = 0.71073 Å
Hall symbol: P 2c -2n	Cell parameters from 9827 reflections
*a* = 16.8879 (5) Å	θ = 2.4–27.7°
*b* = 9.4723 (3) Å	µ = 1.47 mm^−^^1^
*c* = 26.4552 (9) Å	*T* = 100 K
*V* = 4232.0 (2) Å^3^	Prism, colourless
*Z* = 4	0.22 × 0.10 × 0.10 mm

### Data collection

**Table:**

Bruker APEXII CCD diffractometer	9954 independent reflections
Radiation source: fine-focus sealed tube	8605 reflections with *I* > 2σ(*I*)
graphite	*R*_int_ = 0.036
ω and φ scans	θ_max_ = 28.3°, θ_min_ = 1.5°
Absorption correction: multi-scan (*SADABS*; Sheldrick, 1996)	*h* = −21→22
*T*_min_ = 0.738, *T*_max_ = 0.867	*k* = −12→12
31309 measured reflections	*l* = −35→32

### Refinement

**Table:**

Refinement on *F*^2^	Hydrogen site location: inferred from neighbouring sites
Least-squares matrix: full	H atoms treated by a mixture of independent and constrained refinement
*R*[*F*^2^ > 2σ(*F*^2^)] = 0.039	*w* = 1/[σ^2^(*F*_o_^2^) + (0.0392*P*)^2^ + 1.4688*P*] where *P* = (*F*_o_^2^ + 2*F*_c_^2^)/3
*wR*(*F*^2^) = 0.084	(Δ/σ)_max_ = 0.001
*S* = 1.07	Δρ_max_ = 0.56 e Å^−^^3^
9954 reflections	Δρ_min_ = −0.58 e Å^−^^3^
601 parameters	Extinction correction: *SHELXL97* (Sheldrick, 2008), Fc^*^=kFc[1+0.001xFc^2^λ^3^/sin(2θ)]^-1/4^
1 restraint	Extinction coefficient: 0.00024 (8)
Primary atom site location: structure-invariant direct methods	Absolute structure: Flack (1983), 4585 Friedel pairs
Secondary atom site location: difference Fourier map	Flack parameter: 0.383 (7)

### Special details

**Table:**

Geometry. All esds (except the esd in the dihedral angle between two l.s. planes) are estimated using the full covariance matrix. The cell esds are taken into account individually in the estimation of esds in distances, angles and torsion angles; correlations between esds in cell parameters are only used when they are defined by crystal symmetry. An approximate (isotropic) treatment of cell esds is used for estimating esds involving l.s. planes.
Refinement. Refinement of *F*^2^ against ALL reflections. The weighted *R*-factor *wR* and goodness of fit *S* are based on *F*^2^, conventional *R*-factors *R* are based on *F*, with *F* set to zero for negative *F*^2^. The threshold expression of *F*^2^ > σ(*F*^2^) is used only for calculating *R*-factors(gt) *etc*. and is not relevant to the choice of reflections for refinement. *R*-factors based on *F*^2^ are statistically about twice as large as those based on *F*, and *R*- factors based on ALL data will be even larger.

### Fractional atomic coordinates and isotropic or equivalent isotropic displacement parameters (Å^2^)

**Table:**

	*x*	*y*	*z*	*U*_iso_*/*U*_eq_	
Zn1	1.07460 (2)	0.01616 (4)	0.917626 (16)	0.00860 (9)	
N1	1.12568 (18)	−0.1518 (3)	0.96418 (12)	0.0120 (7)	
N2	1.12185 (18)	0.1482 (3)	0.97696 (12)	0.0118 (7)	
N3	0.97800 (19)	−0.0083 (3)	0.97112 (12)	0.0129 (7)	
O1	1.16120 (14)	−0.0640 (3)	0.87080 (10)	0.0118 (5)	
O2	1.21023 (15)	−0.2696 (3)	0.84582 (10)	0.0149 (6)	
O3	1.08723 (14)	0.2045 (3)	0.87911 (10)	0.0109 (5)	
O4	1.14849 (15)	0.4106 (3)	0.88043 (10)	0.0138 (6)	
O5	0.98658 (14)	−0.0531 (3)	0.87180 (10)	0.0127 (6)	
O6	0.85724 (14)	−0.0775 (3)	0.86305 (10)	0.0134 (6)	
C1	1.1941 (2)	−0.0791 (4)	0.98715 (16)	0.0161 (8)	
H1A	1.2341	−0.0596	0.9607	0.019*	
H1B	1.2187	−0.1417	1.0127	0.019*	
C2	1.1699 (2)	0.0601 (4)	1.01240 (15)	0.0174 (8)	
H2A	1.1386	0.0398	1.0432	0.021*	
H2B	1.2179	0.1128	1.0226	0.021*	
C3	1.0484 (2)	0.2058 (4)	1.00086 (15)	0.0134 (8)	
H3A	1.0233	0.2739	0.9774	0.016*	
H3B	1.0629	0.2573	1.0321	0.016*	
C4	0.9891 (2)	0.0898 (4)	1.01387 (15)	0.0169 (9)	
H4A	1.0082	0.0367	1.0437	0.020*	
H4B	0.9376	0.1332	1.0227	0.020*	
C5	0.9838 (2)	−0.1590 (4)	0.98620 (15)	0.0157 (8)	
H5A	0.9663	−0.2188	0.9576	0.019*	
H5B	0.9474	−0.1764	1.0149	0.019*	
C6	1.0663 (2)	−0.2020 (4)	1.00137 (14)	0.0156 (8)	
H6A	1.0787	−0.1621	1.0350	0.019*	
H6B	1.0692	−0.3061	1.0039	0.019*	
C7	1.1508 (2)	−0.2609 (4)	0.92774 (14)	0.0152 (8)	
H7A	1.1064	−0.3272	0.9217	0.018*	
H7B	1.1955	−0.3151	0.9421	0.018*	
C8	1.1760 (2)	−0.1946 (4)	0.87764 (14)	0.0095 (7)	
C9	1.1674 (2)	0.2606 (4)	0.95153 (14)	0.0140 (8)	
H9A	1.2228	0.2292	0.9470	0.017*	
H9B	1.1678	0.3460	0.9731	0.017*	
C10	1.1323 (2)	0.2972 (4)	0.90042 (14)	0.0107 (8)	
C11	0.9056 (2)	0.0144 (4)	0.94157 (15)	0.0128 (8)	
H11A	0.8936	0.1166	0.9399	0.015*	
H11B	0.8605	−0.0336	0.9582	0.015*	
C12	0.9163 (2)	−0.0434 (4)	0.88855 (15)	0.0120 (8)	
Zn2	0.60272 (2)	0.05820 (4)	0.708125 (16)	0.00988 (9)	
N21	0.53471 (18)	−0.0972 (3)	0.66409 (11)	0.0111 (7)	
N22	0.68311 (18)	0.0323 (3)	0.64345 (12)	0.0137 (7)	
N23	0.54514 (19)	0.2003 (3)	0.65352 (12)	0.0141 (7)	
O21	0.52931 (15)	−0.0254 (3)	0.76249 (10)	0.0134 (6)	
O22	0.45361 (15)	−0.2103 (3)	0.78197 (10)	0.0168 (6)	
O23	0.70301 (16)	−0.0144 (3)	0.74292 (10)	0.0160 (6)	
O24	0.83384 (17)	−0.0339 (3)	0.73661 (12)	0.0267 (7)	
O25	0.60456 (15)	0.2484 (3)	0.74679 (10)	0.0142 (6)	
O26	0.55583 (15)	0.4664 (3)	0.74935 (10)	0.0145 (6)	
C21	0.5825 (2)	−0.1491 (4)	0.62115 (14)	0.0129 (8)	
H21A	0.5636	−0.1044	0.5895	0.015*	
H21B	0.5754	−0.2524	0.6178	0.015*	
C22	0.6700 (2)	−0.1160 (4)	0.62845 (16)	0.0162 (8)	
H22A	0.6920	−0.1791	0.6548	0.019*	
H22B	0.6986	−0.1350	0.5965	0.019*	
C23	0.6640 (2)	0.1362 (4)	0.60296 (15)	0.0161 (8)	
H23A	0.6354	0.0882	0.5752	0.019*	
H23B	0.7136	0.1761	0.5891	0.019*	
C24	0.6130 (2)	0.2540 (4)	0.62434 (16)	0.0160 (8)	
H24A	0.6460	0.3145	0.6465	0.019*	
H24B	0.5933	0.3132	0.5962	0.019*	
C25	0.4863 (2)	0.1230 (4)	0.62259 (15)	0.0151 (8)	
H25A	0.5090	0.1028	0.5888	0.018*	
H25B	0.4386	0.1823	0.6179	0.018*	
C26	0.4633 (2)	−0.0149 (4)	0.64821 (15)	0.0132 (8)	
H26A	0.4303	0.0058	0.6783	0.016*	
H26B	0.4312	−0.0725	0.6246	0.016*	
C27	0.5128 (2)	−0.2061 (4)	0.70054 (15)	0.0145 (8)	
H27A	0.5563	−0.2757	0.7034	0.017*	
H27B	0.4650	−0.2564	0.6884	0.017*	
C28	0.4961 (2)	−0.1420 (4)	0.75278 (14)	0.0121 (8)	
C29	0.7628 (2)	0.0498 (4)	0.66439 (15)	0.0175 (9)	
H29A	0.7774	0.1510	0.6637	0.021*	
H29B	0.8013	−0.0023	0.6432	0.021*	
C30	0.7672 (2)	−0.0043 (4)	0.71825 (16)	0.0165 (9)	
C31	0.5101 (2)	0.3140 (4)	0.68376 (14)	0.0140 (8)	
H31A	0.4565	0.2856	0.6950	0.017*	
H31B	0.5048	0.3998	0.6627	0.017*	
C32	0.5604 (2)	0.3469 (4)	0.72938 (15)	0.0140 (8)	
Cl1P	0.90733 (6)	0.49270 (10)	0.94241 (4)	0.0164 (2)	
Cl2P	0.76010 (6)	0.50986 (10)	0.57365 (4)	0.0207 (2)	
Cl3P	0.85405 (6)	0.29914 (10)	0.79612 (4)	0.0198 (2)	
O1P	0.87409 (16)	0.4307 (3)	0.81979 (11)	0.0225 (7)	
O2P	0.78137 (15)	0.2457 (3)	0.81750 (11)	0.0215 (7)	
O3P	0.91798 (15)	0.2025 (3)	0.80605 (11)	0.0199 (6)	
O4P	0.84347 (19)	0.3166 (3)	0.74240 (12)	0.0322 (8)	
O5P	0.98116 (16)	0.4732 (3)	0.91612 (13)	0.0264 (7)	
O6P	0.92441 (19)	0.5250 (3)	0.99436 (12)	0.0300 (8)	
O7P	0.86528 (16)	0.6093 (3)	0.91977 (11)	0.0196 (6)	
O8P	0.86005 (19)	0.3672 (3)	0.93927 (13)	0.0326 (8)	
O9P	0.81100 (17)	0.6321 (3)	0.57245 (13)	0.0307 (7)	
O10P	0.7577 (3)	0.4429 (4)	0.52517 (14)	0.0578 (12)	
O11P	0.68191 (19)	0.5555 (3)	0.58564 (16)	0.0445 (10)	
O12P	0.78848 (18)	0.4105 (3)	0.61010 (12)	0.0303 (8)	
C1S	0.7491 (3)	0.6009 (5)	0.71492 (18)	0.0350 (12)	
H1S1	0.7719	0.5133	0.7016	0.053*	
H1S2	0.7837	0.6804	0.7064	0.053*	
H1S3	0.6967	0.6160	0.6999	0.053*	
O1S	0.74181 (18)	0.5907 (3)	0.76745 (11)	0.0238 (7)	
H1S	0.708 (3)	0.650 (5)	0.7779 (19)	0.036*	
Na1	0.94165 (8)	0.95334 (15)	0.78618 (6)	0.0129 (3)	
Na2	0.84425 (8)	0.67402 (15)	0.83292 (6)	0.0180 (3)	
Na3	0.52011 (8)	0.11140 (16)	0.83393 (6)	0.0175 (3)	
Na4	0.66600 (9)	0.40410 (15)	0.80596 (6)	0.0146 (3)	
Na5	0.74113 (8)	−0.00225 (14)	0.82877 (6)	0.0121 (3)	

### Atomic displacement parameters (Å^2^)

**Table:**

	*U*^11^	*U*^22^	*U*^33^	*U*^12^	*U*^13^	*U*^23^
Zn1	0.00958 (19)	0.00826 (19)	0.0080 (2)	−0.00044 (15)	−0.00002 (19)	−0.00020 (19)
N1	0.0161 (16)	0.0096 (15)	0.0102 (16)	−0.0003 (12)	−0.0014 (13)	−0.0001 (13)
N2	0.0135 (16)	0.0102 (16)	0.0117 (16)	−0.0013 (12)	−0.0008 (13)	−0.0011 (13)
N3	0.0172 (18)	0.0110 (16)	0.0106 (16)	−0.0007 (12)	0.0024 (14)	−0.0027 (13)
O1	0.0138 (13)	0.0109 (13)	0.0106 (13)	0.0039 (10)	0.0041 (11)	0.0003 (11)
O2	0.0156 (14)	0.0128 (14)	0.0164 (14)	0.0043 (10)	0.0033 (12)	−0.0003 (11)
O3	0.0119 (13)	0.0117 (13)	0.0092 (13)	−0.0018 (10)	−0.0016 (11)	0.0001 (11)
O4	0.0160 (14)	0.0092 (13)	0.0162 (14)	−0.0019 (10)	0.0031 (11)	0.0019 (11)
O5	0.0076 (13)	0.0175 (14)	0.0129 (14)	−0.0008 (10)	0.0012 (11)	−0.0014 (11)
O6	0.0137 (13)	0.0096 (13)	0.0169 (14)	−0.0006 (10)	−0.0021 (12)	0.0009 (11)
C1	0.011 (2)	0.021 (2)	0.016 (2)	0.0011 (15)	−0.0043 (16)	0.0070 (17)
C2	0.023 (2)	0.019 (2)	0.010 (2)	−0.0010 (17)	−0.0045 (17)	0.0019 (17)
C3	0.0168 (19)	0.0116 (18)	0.012 (2)	−0.0009 (15)	0.0002 (17)	−0.0036 (15)
C4	0.018 (2)	0.021 (2)	0.012 (2)	−0.0015 (16)	0.0039 (17)	−0.0072 (17)
C5	0.019 (2)	0.019 (2)	0.0092 (19)	−0.0068 (16)	0.0041 (16)	0.0022 (16)
C6	0.023 (2)	0.014 (2)	0.010 (2)	−0.0046 (16)	0.0002 (17)	0.0041 (15)
C7	0.020 (2)	0.0091 (18)	0.016 (2)	0.0005 (14)	0.0023 (17)	−0.0012 (15)
C8	0.0067 (17)	0.0114 (18)	0.0104 (19)	0.0003 (13)	−0.0034 (15)	−0.0009 (15)
C9	0.016 (2)	0.0124 (19)	0.014 (2)	−0.0023 (15)	−0.0031 (16)	−0.0035 (16)
C10	0.0114 (18)	0.0064 (17)	0.0142 (19)	0.0013 (13)	0.0013 (15)	−0.0013 (14)
C11	0.0127 (19)	0.0101 (18)	0.016 (2)	−0.0017 (14)	0.0032 (16)	−0.0029 (16)
C12	0.014 (2)	0.0089 (18)	0.013 (2)	0.0000 (14)	−0.0019 (16)	0.0032 (15)
Zn2	0.0111 (2)	0.0108 (2)	0.0077 (2)	0.00016 (16)	0.00000 (19)	−0.00050 (18)
N21	0.0112 (16)	0.0143 (17)	0.0078 (16)	0.0000 (12)	0.0030 (13)	−0.0013 (13)
N22	0.0101 (16)	0.0163 (16)	0.0148 (17)	−0.0048 (12)	−0.0013 (14)	−0.0020 (14)
N23	0.0176 (17)	0.0144 (16)	0.0103 (16)	−0.0007 (13)	−0.0013 (14)	−0.0003 (14)
O21	0.0161 (14)	0.0144 (14)	0.0095 (14)	−0.0014 (11)	0.0025 (11)	0.0012 (11)
O22	0.0164 (14)	0.0139 (14)	0.0202 (15)	0.0009 (11)	0.0064 (12)	0.0014 (12)
O23	0.0185 (15)	0.0166 (14)	0.0130 (15)	0.0031 (11)	−0.0027 (12)	−0.0022 (12)
O24	0.0146 (15)	0.0318 (18)	0.034 (2)	0.0028 (12)	−0.0097 (14)	−0.0080 (15)
O25	0.0163 (14)	0.0130 (14)	0.0133 (14)	0.0019 (11)	−0.0037 (12)	−0.0006 (11)
O26	0.0136 (14)	0.0102 (13)	0.0197 (15)	−0.0007 (10)	0.0038 (12)	−0.0048 (11)
C21	0.016 (2)	0.0158 (19)	0.0072 (18)	−0.0027 (15)	0.0010 (15)	−0.0052 (15)
C22	0.017 (2)	0.018 (2)	0.014 (2)	0.0015 (16)	0.0035 (17)	−0.0061 (17)
C23	0.018 (2)	0.020 (2)	0.0105 (19)	−0.0059 (16)	0.0012 (17)	0.0003 (17)
C24	0.017 (2)	0.016 (2)	0.014 (2)	−0.0064 (15)	−0.0006 (17)	−0.0004 (17)
C25	0.016 (2)	0.014 (2)	0.015 (2)	−0.0009 (15)	−0.0052 (17)	0.0031 (16)
C26	0.014 (2)	0.0134 (19)	0.0118 (19)	−0.0017 (14)	−0.0033 (16)	−0.0011 (16)
C27	0.0155 (19)	0.0154 (19)	0.013 (2)	−0.0046 (14)	0.0017 (16)	−0.0016 (16)
C28	0.0121 (19)	0.0119 (19)	0.0122 (19)	0.0024 (15)	0.0004 (16)	0.0007 (15)
C29	0.010 (2)	0.028 (2)	0.015 (2)	−0.0014 (16)	−0.0020 (16)	−0.0045 (17)
C30	0.018 (2)	0.0094 (18)	0.022 (2)	−0.0006 (14)	−0.0025 (18)	−0.0039 (16)
C31	0.016 (2)	0.0107 (19)	0.015 (2)	0.0041 (14)	−0.0009 (17)	0.0030 (16)
C32	0.0125 (19)	0.0163 (19)	0.0131 (19)	0.0000 (15)	0.0081 (16)	0.0014 (16)
Cl1P	0.0172 (5)	0.0118 (4)	0.0201 (5)	0.0008 (4)	0.0043 (4)	0.0007 (4)
Cl2P	0.0179 (5)	0.0190 (5)	0.0250 (6)	−0.0022 (4)	−0.0024 (4)	0.0056 (4)
Cl3P	0.0173 (5)	0.0157 (5)	0.0264 (6)	−0.0003 (4)	−0.0004 (4)	−0.0019 (4)
O1P	0.0213 (15)	0.0160 (15)	0.0302 (18)	−0.0028 (12)	−0.0018 (13)	−0.0033 (13)
O2P	0.0113 (15)	0.0157 (14)	0.0376 (19)	−0.0009 (11)	0.0024 (13)	0.0032 (13)
O3P	0.0133 (14)	0.0142 (15)	0.0322 (17)	0.0006 (11)	−0.0025 (13)	−0.0011 (13)
O4P	0.041 (2)	0.0296 (18)	0.0256 (17)	0.0040 (15)	−0.0028 (15)	−0.0033 (15)
O5P	0.0209 (15)	0.0348 (16)	0.0236 (16)	0.0103 (12)	0.0060 (15)	0.0010 (15)
O6P	0.040 (2)	0.0317 (18)	0.0178 (16)	0.0064 (14)	0.0047 (15)	0.0021 (14)
O7P	0.0225 (15)	0.0153 (13)	0.0211 (15)	0.0024 (11)	−0.0027 (14)	0.0028 (13)
O8P	0.0323 (19)	0.0170 (16)	0.048 (2)	−0.0055 (13)	0.0015 (16)	0.0053 (15)
O9P	0.0257 (17)	0.0278 (17)	0.0384 (19)	−0.0113 (13)	−0.0034 (15)	0.0137 (15)
O10P	0.093 (3)	0.054 (3)	0.026 (2)	0.006 (2)	−0.011 (2)	−0.0121 (19)
O11P	0.0185 (17)	0.0303 (19)	0.085 (3)	0.0005 (14)	0.0062 (18)	0.022 (2)
O12P	0.0276 (18)	0.0225 (17)	0.041 (2)	−0.0039 (13)	−0.0087 (15)	0.0138 (14)
C1S	0.041 (3)	0.032 (3)	0.032 (3)	0.005 (2)	−0.002 (2)	0.001 (2)
O1S	0.0243 (17)	0.0277 (17)	0.0193 (16)	0.0010 (13)	0.0058 (14)	−0.0048 (13)
Na1	0.0106 (7)	0.0141 (8)	0.0140 (8)	−0.0015 (6)	−0.0006 (6)	0.0006 (6)
Na2	0.0176 (8)	0.0149 (8)	0.0216 (8)	0.0011 (6)	0.0059 (7)	−0.0020 (7)
Na3	0.0161 (8)	0.0166 (8)	0.0198 (9)	0.0007 (6)	−0.0018 (7)	−0.0064 (7)
Na4	0.0157 (8)	0.0149 (8)	0.0130 (8)	0.0000 (6)	0.0011 (6)	−0.0036 (6)
Na5	0.0136 (8)	0.0104 (7)	0.0124 (8)	−0.0001 (5)	−0.0012 (6)	−0.0002 (6)

### Geometric parameters (Å, °)

**Table:**

Zn1—O5	2.027 (3)	O24—Na5	2.913 (3)
Zn1—O1	2.062 (2)	O25—C32	1.280 (4)
Zn1—O3	2.066 (2)	O25—Na4	2.388 (3)
Zn1—N2	2.160 (3)	O25—Na3	3.005 (3)
Zn1—N3	2.172 (3)	O26—C32	1.251 (4)
Zn1—N1	2.189 (3)	O26—Na1^v^	2.291 (3)
N1—C7	1.475 (5)	O26—Na4	2.460 (3)
N1—C1	1.477 (5)	C21—C22	1.524 (5)
N1—C6	1.483 (5)	C21—H21A	0.9900
N2—C9	1.475 (5)	C21—H21B	0.9900
N2—C3	1.495 (5)	C22—H22A	0.9900
N2—C2	1.495 (5)	C22—H22B	0.9900
N3—C11	1.467 (5)	C23—C24	1.519 (5)
N3—C4	1.476 (5)	C23—H23A	0.9900
N3—C5	1.485 (5)	C23—H23B	0.9900
O1—C8	1.276 (4)	C24—H24A	0.9900
O1—Na4^i^	2.289 (3)	C24—H24B	0.9900
O2—C8	1.244 (4)	C25—C26	1.523 (5)
O2—Na5^ii^	2.268 (3)	C25—H25A	0.9900
O2—Na2^i^	2.461 (3)	C25—H25B	0.9900
O3—C10	1.291 (4)	C26—H26A	0.9900
O3—Na3^i^	2.399 (3)	C26—H26B	0.9900
O3—Na4^i^	2.564 (3)	C27—C28	1.536 (5)
O4—C10	1.228 (4)	C27—H27A	0.9900
O4—Na5^i^	2.252 (3)	C27—H27B	0.9900
O4—Na3^i^	2.501 (3)	C29—C30	1.516 (6)
O5—C12	1.271 (4)	C29—H29A	0.9900
O5—Na1^iii^	2.390 (3)	C29—H29B	0.9900
O6—C12	1.246 (4)	C30—Na5	2.957 (4)
O6—Na5	2.275 (3)	C31—C32	1.508 (5)
O6—Na2^iii^	2.495 (3)	C31—H31A	0.9900
O6—Na1^iii^	2.501 (3)	C31—H31B	0.9900
C1—C2	1.533 (6)	C32—Na4	2.753 (4)
C1—H1A	0.9900	Cl1P—O8P	1.435 (3)
C1—H1B	0.9900	Cl1P—O6P	1.437 (3)
C2—H2A	0.9900	Cl1P—O5P	1.440 (3)
C2—H2B	0.9900	Cl1P—O7P	1.444 (3)
C3—C4	1.527 (5)	Cl2P—O11P	1.425 (3)
C3—H3A	0.9900	Cl2P—O12P	1.430 (3)
C3—H3B	0.9900	Cl2P—O10P	1.431 (4)
C4—H4A	0.9900	Cl2P—O9P	1.442 (3)
C4—H4B	0.9900	Cl3P—O1P	1.435 (3)
C5—C6	1.507 (5)	Cl3P—O3P	1.440 (3)
C5—H5A	0.9900	Cl3P—O4P	1.442 (3)
C5—H5B	0.9900	Cl3P—O2P	1.443 (3)
C6—H6A	0.9900	Cl3P—Na3^i^	3.0957 (17)
C6—H6B	0.9900	Cl3P—Na4	3.3378 (18)
C7—C8	1.527 (5)	O1P—Na2	2.385 (3)
C7—H7A	0.9900	O1P—Na3^i^	2.526 (3)
C7—H7B	0.9900	O2P—Na5	2.463 (3)
C8—Na2^i^	3.083 (4)	O2P—Na4	2.478 (3)
C9—C10	1.516 (5)	O3P—Na1^iii^	2.451 (3)
C9—H9A	0.9900	O3P—Na3^i^	2.574 (3)
C9—H9B	0.9900	O5P—Na3^i^	2.409 (4)
C10—Na3^i^	2.727 (4)	O7P—Na2	2.404 (3)
C11—C12	1.516 (5)	C1S—O1S	1.398 (6)
C11—H11A	0.9900	C1S—H1S1	0.9800
C11—H11B	0.9900	C1S—H1S2	0.9800
C12—Na1^iii^	2.742 (4)	C1S—H1S3	0.9800
Zn2—O23	2.047 (3)	O1S—Na4	2.409 (3)
Zn2—O21	2.057 (3)	O1S—Na2	2.572 (3)
Zn2—O25	2.072 (3)	O1S—H1S	0.85 (5)
Zn2—N22	2.198 (3)	Na1—O24^vi^	2.247 (3)
Zn2—N21	2.201 (3)	Na1—O26^vii^	2.291 (3)
Zn2—N23	2.201 (3)	Na1—O22^i^	2.313 (3)
Zn2—Na3	3.6439 (16)	Na1—O5^vi^	2.390 (3)
N21—C27	1.460 (5)	Na1—O3P^vi^	2.451 (3)
N21—C21	1.477 (5)	Na1—O6^vi^	2.501 (3)
N21—C26	1.496 (5)	Na1—C12^vi^	2.742 (4)
N22—C29	1.465 (5)	Na2—O22^i^	2.312 (3)
N22—C22	1.476 (5)	Na2—O2^iv^	2.461 (3)
N22—C23	1.490 (5)	Na2—O6^vi^	2.495 (3)
N23—C31	1.467 (5)	Na2—C8^iv^	3.083 (4)
N23—C24	1.473 (5)	Na3—O3^iv^	2.399 (3)
N23—C25	1.480 (5)	Na3—O5P^iv^	2.409 (4)
O21—C28	1.265 (4)	Na3—O4^iv^	2.501 (3)
O21—Na3	2.297 (3)	Na3—O1P^iv^	2.526 (3)
O22—C28	1.237 (4)	Na3—O3P^iv^	2.574 (3)
O22—Na2^iv^	2.312 (3)	Na3—C10^iv^	2.727 (4)
O22—Na1^iv^	2.313 (3)	Na3—Cl3P^iv^	3.0957 (17)
O23—C30	1.269 (5)	Na4—O1^iv^	2.289 (3)
O23—Na5	2.364 (3)	Na4—O3^iv^	2.564 (3)
O24—C30	1.257 (5)	Na5—O4^iv^	2.252 (3)
O24—Na1^iii^	2.247 (3)	Na5—O2^viii^	2.268 (3)
			
O5—Zn1—O1	92.38 (10)	N23—C24—H24A	109.1
O5—Zn1—O3	93.47 (10)	C23—C24—H24A	109.1
O1—Zn1—O3	87.04 (10)	N23—C24—H24B	109.1
O5—Zn1—N2	153.25 (11)	C23—C24—H24B	109.1
O1—Zn1—N2	112.82 (11)	H24A—C24—H24B	107.8
O3—Zn1—N2	79.63 (11)	N23—C25—C26	110.5 (3)
O5—Zn1—N3	78.72 (11)	N23—C25—H25A	109.6
O1—Zn1—N3	152.26 (11)	C26—C25—H25A	109.6
O3—Zn1—N3	119.42 (11)	N23—C25—H25B	109.6
N2—Zn1—N3	82.29 (12)	C26—C25—H25B	109.6
O5—Zn1—N1	112.96 (11)	H25A—C25—H25B	108.1
O1—Zn1—N1	77.94 (11)	N21—C26—C25	111.5 (3)
O3—Zn1—N1	149.83 (11)	N21—C26—H26A	109.3
N2—Zn1—N1	82.32 (12)	C25—C26—H26A	109.3
N3—Zn1—N1	81.47 (12)	N21—C26—H26B	109.3
O5—Zn1—Na4^i^	83.44 (8)	C25—C26—H26B	109.3
O1—Zn1—Na4^i^	40.66 (8)	H26A—C26—H26B	108.0
O3—Zn1—Na4^i^	48.41 (7)	N21—C27—C28	111.2 (3)
N2—Zn1—Na4^i^	109.47 (9)	N21—C27—H27A	109.4
N3—Zn1—Na4^i^	157.82 (9)	C28—C27—H27A	109.4
N1—Zn1—Na4^i^	117.97 (9)	N21—C27—H27B	109.4
C7—N1—C1	111.8 (3)	C28—C27—H27B	109.4
C7—N1—C6	113.8 (3)	H27A—C27—H27B	108.0
C1—N1—C6	113.9 (3)	O22—C28—O21	126.0 (4)
C7—N1—Zn1	104.7 (2)	O22—C28—C27	117.5 (3)
C1—N1—Zn1	101.6 (2)	O21—C28—C27	116.5 (3)
C6—N1—Zn1	109.9 (2)	N22—C29—C30	111.2 (3)
C9—N2—C3	111.2 (3)	N22—C29—H29A	109.4
C9—N2—C2	114.0 (3)	C30—C29—H29A	109.4
C3—N2—C2	112.9 (3)	N22—C29—H29B	109.4
C9—N2—Zn1	106.2 (2)	C30—C29—H29B	109.4
C3—N2—Zn1	102.3 (2)	H29A—C29—H29B	108.0
C2—N2—Zn1	109.4 (2)	O24—C30—O23	123.3 (4)
C11—N3—C4	114.9 (3)	O24—C30—C29	118.8 (4)
C11—N3—C5	109.8 (3)	O23—C30—C29	117.8 (3)
C4—N3—C5	113.0 (3)	O24—C30—Na5	75.7 (2)
C11—N3—Zn1	105.3 (2)	O23—C30—Na5	50.56 (19)
C4—N3—Zn1	109.7 (2)	C29—C30—Na5	156.9 (3)
C5—N3—Zn1	103.2 (2)	N23—C31—C32	111.2 (3)
C8—O1—Zn1	114.3 (2)	N23—C31—H31A	109.4
C8—O1—Na4^i^	137.8 (2)	C32—C31—H31A	109.4
Zn1—O1—Na4^i^	103.41 (11)	N23—C31—H31B	109.4
C8—O2—Na5^ii^	141.8 (2)	C32—C31—H31B	109.4
C8—O2—Na2^i^	108.1 (2)	H31A—C31—H31B	108.0
Na5^ii^—O2—Na2^i^	96.39 (10)	O26—C32—O25	122.9 (4)
C10—O3—Zn1	115.6 (2)	O26—C32—C31	119.3 (3)
C10—O3—Na3^i^	90.2 (2)	O25—C32—C31	117.7 (3)
Zn1—O3—Na3^i^	145.57 (12)	O26—C32—Na4	63.3 (2)
C10—O3—Na4^i^	107.2 (2)	O25—C32—Na4	60.11 (19)
Zn1—O3—Na4^i^	94.54 (9)	C31—C32—Na4	173.9 (3)
Na3^i^—O3—Na4^i^	99.25 (10)	O8P—Cl1P—O6P	110.1 (2)
C10—O4—Na5^i^	138.8 (2)	O8P—Cl1P—O5P	110.33 (19)
C10—O4—Na3^i^	86.9 (2)	O6P—Cl1P—O5P	108.4 (2)
Na5^i^—O4—Na3^i^	109.63 (12)	O8P—Cl1P—O7P	109.66 (18)
C12—O5—Zn1	116.9 (2)	O6P—Cl1P—O7P	109.42 (18)
C12—O5—Na1^iii^	91.8 (2)	O5P—Cl1P—O7P	108.90 (18)
Zn1—O5—Na1^iii^	142.31 (13)	O11P—Cl2P—O12P	111.1 (2)
C12—O6—Na5	145.5 (2)	O11P—Cl2P—O10P	107.9 (3)
C12—O6—Na2^iii^	119.2 (2)	O12P—Cl2P—O10P	108.8 (2)
Na5—O6—Na2^iii^	95.30 (10)	O11P—Cl2P—O9P	108.29 (19)
C12—O6—Na1^iii^	87.4 (2)	O12P—Cl2P—O9P	110.07 (19)
Na5—O6—Na1^iii^	97.50 (11)	O10P—Cl2P—O9P	110.7 (2)
Na2^iii^—O6—Na1^iii^	84.29 (9)	O1P—Cl3P—O3P	107.20 (16)
N1—C1—C2	111.8 (3)	O1P—Cl3P—O4P	111.07 (19)
N1—C1—H1A	109.3	O3P—Cl3P—O4P	110.22 (18)
C2—C1—H1A	109.3	O1P—Cl3P—O2P	109.51 (18)
N1—C1—H1B	109.3	O3P—Cl3P—O2P	110.08 (17)
C2—C1—H1B	109.3	O4P—Cl3P—O2P	108.74 (18)
H1A—C1—H1B	107.9	O1P—Cl3P—Na3^i^	53.68 (12)
N2—C2—C1	110.6 (3)	O3P—Cl3P—Na3^i^	55.65 (11)
N2—C2—H2A	109.5	O4P—Cl3P—Na3^i^	113.53 (14)
C1—C2—H2A	109.5	O2P—Cl3P—Na3^i^	137.71 (13)
N2—C2—H2B	109.5	O1P—Cl3P—Na4	86.08 (12)
C1—C2—H2B	109.5	O3P—Cl3P—Na4	152.71 (13)
H2A—C2—H2B	108.1	O4P—Cl3P—Na4	85.68 (13)
N2—C3—C4	112.2 (3)	O2P—Cl3P—Na4	42.67 (11)
N2—C3—H3A	109.2	Na3^i^—Cl3P—Na4	139.04 (5)
C4—C3—H3A	109.2	Cl3P—O1P—Na2	148.54 (18)
N2—C3—H3B	109.2	Cl3P—O1P—Na3^i^	99.08 (15)
C4—C3—H3B	109.2	Na2—O1P—Na3^i^	109.71 (11)
H3A—C3—H3B	107.9	Cl3P—O2P—Na5	128.06 (16)
N3—C4—C3	111.3 (3)	Cl3P—O2P—Na4	114.08 (15)
N3—C4—H4A	109.4	Na5—O2P—Na4	112.04 (11)
C3—C4—H4A	109.4	Cl3P—O3P—Na1^iii^	134.07 (16)
N3—C4—H4B	109.4	Cl3P—O3P—Na3^i^	96.85 (13)
C3—C4—H4B	109.4	Na1^iii^—O3P—Na3^i^	127.71 (11)
H4A—C4—H4B	108.0	Cl1P—O5P—Na3^i^	135.67 (19)
N3—C5—C6	113.1 (3)	Cl1P—O7P—Na2	131.63 (17)
N3—C5—H5A	109.0	O1S—C1S—H1S1	109.5
C6—C5—H5A	109.0	O1S—C1S—H1S2	109.5
N3—C5—H5B	109.0	H1S1—C1S—H1S2	109.5
C6—C5—H5B	109.0	O1S—C1S—H1S3	109.5
H5A—C5—H5B	107.8	H1S1—C1S—H1S3	109.5
N1—C6—C5	111.2 (3)	H1S2—C1S—H1S3	109.5
N1—C6—H6A	109.4	C1S—O1S—Na4	121.1 (3)
C5—C6—H6A	109.4	C1S—O1S—Na2	126.1 (3)
N1—C6—H6B	109.4	Na4—O1S—Na2	107.33 (12)
C5—C6—H6B	109.4	C1S—O1S—H1S	110 (3)
H6A—C6—H6B	108.0	Na4—O1S—H1S	90 (3)
N1—C7—C8	111.1 (3)	Na2—O1S—H1S	91 (3)
N1—C7—H7A	109.4	O24^vi^—Na1—O26^vii^	114.58 (12)
C8—C7—H7A	109.4	O24^vi^—Na1—O22^i^	95.52 (12)
N1—C7—H7B	109.4	O26^vii^—Na1—O22^i^	103.68 (11)
C8—C7—H7B	109.4	O24^vi^—Na1—O5^vi^	144.28 (12)
H7A—C7—H7B	108.0	O26^vii^—Na1—O5^vi^	98.30 (10)
O2—C8—O1	123.3 (3)	O22^i^—Na1—O5^vi^	89.56 (11)
O2—C8—C7	118.9 (3)	O24^vi^—Na1—O3P^vi^	86.63 (11)
O1—C8—C7	117.8 (3)	O26^vii^—Na1—O3P^vi^	84.76 (10)
O2—C8—Na2^i^	49.36 (18)	O22^i^—Na1—O3P^vi^	169.35 (12)
O1—C8—Na2^i^	93.7 (2)	O5^vi^—Na1—O3P^vi^	82.72 (10)
C7—C8—Na2^i^	128.1 (2)	O24^vi^—Na1—O6^vi^	91.09 (11)
N2—C9—C10	111.6 (3)	O26^vii^—Na1—O6^vi^	149.84 (11)
N2—C9—H9A	109.3	O22^i^—Na1—O6^vi^	88.43 (10)
C10—C9—H9A	109.3	O5^vi^—Na1—O6^vi^	53.64 (9)
N2—C9—H9B	109.3	O3P^vi^—Na1—O6^vi^	81.09 (10)
C10—C9—H9B	109.3	O22^i^—Na2—O1P	83.69 (11)
H9A—C9—H9B	108.0	O22^i^—Na2—O7P	118.49 (11)
O4—C10—O3	122.5 (3)	O1P—Na2—O7P	82.05 (11)
O4—C10—C9	119.8 (3)	O22^i^—Na2—O2^iv^	139.51 (11)
O3—C10—C9	117.7 (3)	O1P—Na2—O2^iv^	124.75 (11)
O4—C10—Na3^i^	66.3 (2)	O7P—Na2—O2^iv^	95.57 (10)
O3—C10—Na3^i^	61.58 (18)	O22^i^—Na2—O6^vi^	88.60 (10)
C9—C10—Na3^i^	156.8 (3)	O1P—Na2—O6^vi^	160.03 (11)
N3—C11—C12	109.9 (3)	O7P—Na2—O6^vi^	85.54 (10)
N3—C11—H11A	109.7	O2^iv^—Na2—O6^vi^	71.91 (9)
C12—C11—H11A	109.7	O22^i^—Na2—O1S	100.97 (11)
N3—C11—H11B	109.7	O1P—Na2—O1S	75.38 (11)
C12—C11—H11B	109.7	O7P—Na2—O1S	131.66 (11)
H11A—C11—H11B	108.2	O2^iv^—Na2—O1S	65.65 (10)
O6—C12—O5	122.7 (4)	O6^vi^—Na2—O1S	124.31 (11)
O6—C12—C11	119.9 (3)	O21—Na3—O5P^iv^	125.00 (12)
O5—C12—C11	117.4 (3)	O3^iv^—Na3—O5P^iv^	85.47 (10)
O6—C12—Na1^iii^	65.6 (2)	O21—Na3—O4^iv^	107.40 (10)
O5—C12—Na1^iii^	60.57 (19)	O3^iv^—Na3—O4^iv^	53.55 (8)
C11—C12—Na1^iii^	159.4 (2)	O5P^iv^—Na3—O4^iv^	76.41 (10)
O23—Zn2—O21	93.15 (11)	O21—Na3—O1P^iv^	81.67 (11)
O23—Zn2—O25	93.30 (10)	O3^iv^—Na3—O1P^iv^	130.54 (10)
O21—Zn2—O25	89.92 (10)	O5P^iv^—Na3—O1P^iv^	79.32 (11)
O23—Zn2—N22	78.54 (11)	O4^iv^—Na3—O1P^iv^	154.94 (12)
O21—Zn2—N22	150.85 (11)	O21—Na3—O3P^iv^	101.30 (11)
O25—Zn2—N22	118.18 (11)	O3^iv^—Na3—O3P^iv^	87.80 (9)
O23—Zn2—N21	116.44 (11)	O5P^iv^—Na3—O3P^iv^	107.67 (11)
O21—Zn2—N21	78.35 (11)	O4^iv^—Na3—O3P^iv^	141.14 (10)
O25—Zn2—N21	148.30 (11)	O1P^iv^—Na3—O3P^iv^	53.95 (9)
N22—Zn2—N21	80.53 (11)	O21—Na3—C10^iv^	131.52 (12)
O23—Zn2—N23	150.03 (12)	O3^iv^—Na3—C10^iv^	28.26 (9)
O21—Zn2—N23	115.34 (11)	O5P^iv^—Na3—C10^iv^	73.33 (11)
O25—Zn2—N23	78.41 (11)	O4^iv^—Na3—C10^iv^	26.73 (9)
N22—Zn2—N23	80.23 (12)	O1P^iv^—Na3—C10^iv^	145.51 (12)
N21—Zn2—N23	80.27 (12)	O3P^iv^—Na3—C10^iv^	115.66 (11)
O23—Zn2—Na3	87.28 (8)	O21—Na3—O25	65.18 (9)
O21—Zn2—Na3	35.31 (8)	O3^iv^—Na3—O25	81.04 (9)
O25—Zn2—Na3	55.57 (8)	O5P^iv^—Na3—O25	164.93 (10)
N22—Zn2—Na3	164.36 (9)	O4^iv^—Na3—O25	90.11 (9)
N21—Zn2—Na3	112.10 (8)	O1P^iv^—Na3—O25	114.70 (10)
N23—Zn2—Na3	110.23 (9)	O3P^iv^—Na3—O25	78.61 (9)
C27—N21—C21	114.3 (3)	C10^iv^—Na3—O25	91.59 (10)
C27—N21—C26	110.4 (3)	O21—Na3—Cl3P^iv^	87.13 (8)
C21—N21—C26	113.4 (3)	O3^iv^—Na3—Cl3P^iv^	112.97 (8)
C27—N21—Zn2	104.8 (2)	O5P^iv^—Na3—Cl3P^iv^	97.78 (9)
C21—N21—Zn2	110.2 (2)	O4^iv^—Na3—Cl3P^iv^	165.21 (9)
C26—N21—Zn2	102.8 (2)	O1P^iv^—Na3—Cl3P^iv^	27.24 (7)
C29—N22—C22	110.3 (3)	O3P^iv^—Na3—Cl3P^iv^	27.50 (6)
C29—N22—C23	113.3 (3)	C10^iv^—Na3—Cl3P^iv^	138.69 (9)
C22—N22—C23	113.7 (3)	O25—Na3—Cl3P^iv^	93.66 (7)
C29—N22—Zn2	105.1 (2)	O1^iv^—Na4—O25	152.16 (11)
C22—N22—Zn2	102.9 (2)	O1^iv^—Na4—O1S	81.39 (10)
C23—N22—Zn2	110.6 (2)	O25—Na4—O1S	114.02 (11)
C31—N23—C24	110.2 (3)	O1^iv^—Na4—O26	105.71 (10)
C31—N23—C25	113.2 (3)	O25—Na4—O26	54.58 (9)
C24—N23—C25	113.8 (3)	O1S—Na4—O26	88.20 (11)
C31—N23—Zn2	105.6 (2)	O1^iv^—Na4—O2P	109.63 (11)
C24—N23—Zn2	102.2 (2)	O25—Na4—O2P	92.77 (10)
C25—N23—Zn2	110.9 (2)	O1S—Na4—O2P	94.51 (11)
C28—O21—Zn2	117.5 (2)	O26—Na4—O2P	144.57 (11)
C28—O21—Na3	129.0 (2)	O1^iv^—Na4—O3^iv^	71.43 (9)
Zn2—O21—Na3	113.50 (12)	O25—Na4—O3^iv^	91.22 (10)
C28—O22—Na2^iv^	138.5 (2)	O1S—Na4—O3^iv^	152.81 (11)
C28—O22—Na1^iv^	127.0 (2)	O26—Na4—O3^iv^	99.40 (10)
Na2^iv^—O22—Na1^iv^	92.88 (11)	O2P—Na4—O3^iv^	94.12 (10)
C30—O23—Zn2	116.8 (2)	O1^iv^—Na4—C32	131.19 (12)
C30—O23—Na5	104.9 (2)	O25—Na4—C32	27.70 (10)
Zn2—O23—Na5	129.88 (13)	O1S—Na4—C32	100.22 (12)
C30—O24—Na1^iii^	164.3 (3)	O26—Na4—C32	27.03 (10)
C30—O24—Na5	79.6 (2)	O2P—Na4—C32	118.75 (12)
Na1^iii^—O24—Na5	87.28 (11)	O3^iv^—Na4—C32	98.08 (11)
C32—O25—Zn2	116.6 (2)	O1^iv^—Na4—Cl3P	106.80 (8)
C32—O25—Na4	92.2 (2)	O25—Na4—Cl3P	100.27 (8)
Zn2—O25—Na4	150.14 (13)	O1S—Na4—Cl3P	71.34 (8)
C32—O25—Na3	108.3 (2)	O26—Na4—Cl3P	138.03 (9)
Zn2—O25—Na3	89.79 (9)	O2P—Na4—Cl3P	23.25 (7)
Na4—O25—Na3	88.28 (9)	O3^iv^—Na4—Cl3P	115.65 (7)
C32—O26—Na1^v^	122.2 (2)	C32—Na4—Cl3P	120.02 (9)
C32—O26—Na4	89.6 (2)	O4^iv^—Na5—O2^viii^	94.98 (11)
Na1^v^—O26—Na4	117.22 (12)	O4^iv^—Na5—O6	118.54 (12)
N21—C21—C22	111.3 (3)	O2^viii^—Na5—O6	79.66 (10)
N21—C21—H21A	109.4	O4^iv^—Na5—O23	114.38 (11)
C22—C21—H21A	109.4	O2^viii^—Na5—O23	94.71 (11)
N21—C21—H21B	109.4	O6—Na5—O23	127.05 (11)
C22—C21—H21B	109.4	O4^iv^—Na5—O2P	84.12 (10)
H21A—C21—H21B	108.0	O2^viii^—Na5—O2P	174.83 (12)
N22—C22—C21	112.1 (3)	O6—Na5—O2P	96.27 (10)
N22—C22—H22A	109.2	O23—Na5—O2P	90.30 (11)
C21—C22—H22A	109.2	O4^iv^—Na5—O24	157.10 (11)
N22—C22—H22B	109.2	O2^viii^—Na5—O24	101.07 (10)
C21—C22—H22B	109.2	O6—Na5—O24	80.68 (10)
H22A—C22—H22B	107.9	O23—Na5—O24	48.48 (9)
N22—C23—C24	109.9 (3)	O2P—Na5—O24	81.29 (10)
N22—C23—H23A	109.7	O4^iv^—Na5—C30	134.93 (12)
C24—C23—H23A	109.7	O2^viii^—Na5—C30	102.99 (11)
N22—C23—H23B	109.7	O6—Na5—C30	105.29 (11)
C24—C23—H23B	109.7	O23—Na5—C30	24.50 (10)
H23A—C23—H23B	108.2	O2P—Na5—C30	81.10 (11)
N23—C24—C23	112.5 (3)	O24—Na5—C30	24.72 (9)

Symmetry codes: (i) *x*+1/2, −*y*+1/2, *z*; (ii) *x*+1/2, −*y*−1/2, *z*; (iii) *x*, *y*−1, *z*; (iv) *x*−1/2, −*y*+1/2, *z*; (v) *x*−1/2, −*y*+3/2, *z*; (vi) *x*, *y*+1, *z*; (vii) *x*+1/2, −*y*+3/2, *z*; (viii) *x*−1/2, −*y*−1/2, *z*.
